# Effect of Akimbo versus Raised Arm Positioning on Breast and Cardiopulmonary Dosimetry in Pediatric Hodgkin Lymphoma

**DOI:** 10.3389/fonc.2016.00176

**Published:** 2016-07-26

**Authors:** Kyle A. Denniston, Vivek Verma, Abhijeet R. Bhirud, Nathan R. Bennion, Chi Lin

**Affiliations:** ^1^Department of Radiation Oncology, St. Peter’s Health Partners, Albany, NY, USA; ^2^Department of Radiation Oncology, University of Nebraska Medical Center, Omaha, NE, USA

**Keywords:** Hodgkin lymphoma, pediatric cancer, secondary malignancy, dosimetry, radiotherapy

## Abstract

**Purpose:**

In pediatric Hodgkin lymphoma (HL), radiotherapy (RT)-related late toxicities are a prime concern during treatment planning. This is the first study to examine whether arm positioning (raised versus akimbo) result in differential cardiopulmonary and breast doses in patients undergoing mediastinal RT.

**Methods:**

Two treatment plans were made for each patient (akimbo/arms raised); treatment was per Children’s Oncology Group AHOD0031 protocol, including AP/PA fields. The anterior midline T6–T7 disk space was used as an anatomic reference of “midline.” Heart/lungs were contoured for each setup. For females, breasts were also contoured and nipple positions identified. Volumetric centers of contoured organs were defined and three-dimensional distances from “midline” were computed. Analyzed dosimetric parameters included V5 (volume receiving ≥5 Gy), V10, V15, V20, and mean dose. Statistics were performed using the Mann–Whitney test.

**Results:**

Fifteen (6 females, 9 males) pediatric HL patients treated with mediastinal RT were analyzed. The median lateral distance from the breast center/nipple to “midline” with arms akimbo was larger than that with arms raised (8.6 vs. 7.7 cm left breast, *p* = 0.04; 10.7 vs. 9.2 cm left nipple, *p* = 0.04; 8.7 vs. 7.0 cm right breast, *p* = 0.004; 9.9 vs. 7.9 cm right nipple, *p* = 0.007). Raised arm position was associated with a median 2.8/3.0 cm decrease in breast/nipple separation, respectively. There were no significant differences in craniocaudal breast/nipple position based on arm positioning (*p* > 0.05). Increasing breast volume was correlated with larger arm position-related changes in breast/nipple separation (*r* = 0.74, *p* = 0.06/*r* = 0.85, *p* = 0.02). Akimbo positioning lowered median breast V5, V10, V15, and mean dose (*p* < 0.05), with no differences observed in patients with both mediastinal and axillary disease for any parameters (*p* > 0.05). Arm position had no significant effect on cardiopulmonary doses.

**Conclusion:**

Akimbo arm positioning may be advantageous to decrease breast doses in female pediatric HL patients undergoing mediastinal RT, especially in the absence of axillary disease.

## Introduction

Multimodality therapy, namely chemotherapy and radiotherapy (RT), remains the primary treatment of choice for most children and adolescents with Hodgkin lymphoma (HL). With this approach, over 95% of favorable subgroup patients can be long-term survivors (1). Consequently, this high cure rate warrants efforts to reduce treatment-related late morbidities. With regard to RT, late effects are related to exposure of normal tissues [organs at risk (OARs)]. For instance, mediastinal irradiation has been associated with late cardiovascular (2) and pulmonary toxicity (3), along with increased risks of secondary malignancies, including neoplasms of the thyroid, breast, and lung (4–6).

Despite major RT paradigm shifts to smaller treatment volumes and lower doses, long-term sequelae remain a primary concern when treating children and adolescents with RT, and any possible therapeutic adaptation to decrease the risk of such serious complications becomes paramount in such a curable disease. One such approach that has been relatively understudied involves patient positioning. Specifically, arm positioning for mediastinal RT typically follows one of two patterns: with the patient’s arms raised above the head, or at the patient’s sides, termed the akimbo position. Using these approaches, doses to cardiopulmonary OARs as well as breast tissue have, heretofore, not been characterized; furthermore, in some academic and community practices, arm positioning technique in these patients is simply based on physician preference. This study is the first to date to quantify the impact of arm position on spatial and dosimetric parameters for the heart, lungs, and breast tissue in pediatric HL patients.

## Materials and Methods

### Treatment Planning and Dosimetry

After Institutional Review Board and Ethics Committee approval at the University of Nebraska Medical Center, we retrospectively identified and reviewed the treatment plans of pediatric HL patients treated with mediastinal RT at our institution from 2008 to 2013. All patients received treatment planning (including technique, field design/borders, and blocking) and delivery per the contemporary children’s oncology group (COG) protocol AHOD0031, delivering involved field RT to a total dose of 21 Gy in 14 fractions of 1.5 Gy each (7). Prior to computed tomography (CT) simulation, all patients underwent staging diagnostic CT and/or positron emission tomography (PET)-CT (in the arms raised position). Simulation, performed supine and with intravenous contrast, included deep inspiration breath hold (DIBH) technique in six patients. In total, each patient had two complete treatment plans made, differing only in arm position. OARs were manually segmented on a slice-by-slice basis (per AHOD0031 and RTOG guidelines, when applicable) and organ volumes were recorded, including lungs, heart, and bilateral breasts (in female patients). After fusion of pre- and post-chemotherapy PET-CT, target volumes were contoured and fields designed per the protocol in each simulation CT scan. After two treatment plans per patient were made (without heterogeneity corrections per protocol), multiple dosimetric quantities were then recorded for both plans, including V5 [volume (cubic centimeters) receiving ≥5 Gy], V10, V15, V20, and mean dose for the lungs, breasts, and heart.

### Spatial Position Evaluation

In addition to dosimetric calculations, we sought to quantify the spatial position of each OAR. Organ position was quantified by calculating the three-dimensional distance [superior–inferior (*z*-axis), lateral (*x*-axis), and anterior–posterior (*y*-axis)] from the anatomic central point (centroid) of each OAR volume to a fixed reference point, which was placed at the anterior midline T6–7 disk space. This point was chosen because it could be reliably demonstrated and reproduced on available axial image sequences for all patients, and because it served as a rigid marker of the anatomic “midline” of the mediastinum (and thus the treatment field given the AP/PA RT technique). Consequently, for female patients, the distance (in three dimensions) between the centroids of the right and left breasts was calculated relative to the midline reference point (the lateral/*x*-axis measurements of this parameter was termed the separation distance). This was also performed for three-dimensional distance from each nipple to the reference point.

### Statistical Analysis

Statistics, performed using SAS software version 9.4 (Cary, NC, USA). Both spatial and dosimetric parameters were performed using the Mann–Whitney test. The Pearson product–moment correlation coefficient was calculated to evaluate the relationship of breast size and arm position-related changes in breast separation. Paired Student’s *t*-tests were utilized to compare differences in breast volume based on positioning.

## Results

### Patient Population

During the study period, 27 consecutively treated pediatric HL patients were evaluated. Of these, 21 received mediastinal RT. Six patients were unable to be analyzed owing to inaccessible or corrupted/distorted data. The study group therefore consisted of 15 patients (6 females, 9 males). Selected clinical characteristics of the patient population are shown in Table 1.

**Table 1:** **Clinical characteristics of the study population**.

Patient	Gender	Age	BMI (kg/m^2^)	Histology	Bulky disease^a^	Involved supradiaphragmatic nodal sites (in addition to mediastinum)
1	Male	12	27.3	NS	No	Bilateral cervical, bilateral axillary
2	Female	13	22.7	NS	Yes	Left cervical, left axillary
3	Male	13	21.4	NS	No	Bilateral cervical, bilateral infraclavicular, bilateral axillary
4	Female	13	23.7	NS	No	Bilateral cervical, left axillary
5	Male	15	36.3	NS	Yes	Left cervical
6	Female	15	29.3	NS	No	Bilateral cervical
7	Male	15	21.9	NS	No	Right cervical
8	Male	15	–	NS	Yes	Bilateral cervical
9	Female	16	30.5	NS	No	Bilateral cervical
10	Male	16	20.4	NS	Yes	Bilateral cervical
11	Male	18	25.7	NS	No	Bilateral cervical
12	Female	17	22.4	NS	No	Right cervical
13	Female	12	22.9	NS	Yes	Bilateral cervical, bilateral axillary
14	Male	13	23.1	NS	Yes	Bilateral cervical
15	Male	15	25.3	NS	No	Bilateral cervical, right axillary

*BMI, body mass index; NS, nodular sclerosis*.

*^a^Definition per AHOD0031*.

### Breast Spatial Analysis

The median breast volume for female patients on the akimbo simulation CT was 454.0 (range, 123.8–1063.1) cm^3^ on the left and 443.1 (range, 116.8–982.4) cm^3^ on the right. Corresponding numbers were similar on the raised arm CT, 469.1 (range, 107.4–1017.1) cm^3^ and 425.2 (range, 104.8–930.6) cm^3^, respectively. Neither of these were statistically different between positioning groups (*p* = 0.681 and *p* = 0.346, respectively).

Spatial measurements are denoted in Table 2. Arm positioning most prominently impacted the lateral measurements; the median craniocaudal distance from the breast centroid/nipple to the T6–7 reference point was not significantly affected by arm position for either breast (*p* > 0.05 for all). The median lateral distance from the left breast centroid to midline was 8.6 and 7.7 cm with arms akimbo and raised, respectively (*p* = 0.04). Corresponding figures from the left nipple to midline were 10.7 and 9.2 cm with arms akimbo and raised, respectively (*p* = 0.04). Similar results were seen for the right breast centroid to midline (9.9 cm akimbo vs. 7.9 cm raised, *p* = 0.004) and right nipple to midline (8.7 cm akimbo vs. 7.0 cm raised, *p* = 0.007).

**Table 2:** **Arm position-related changes in breast position and separation**.

Distance (cm)		Arm position		*p*-Value	Change in separation distance (cm)
		Raised	Akimbo		
Nipple to midline	Left	9.2 (6.1–10.3)	10.7 (9.8–16.3)	0.04	2.8 (2.3–12.5)
	Right	7.9 (7.0–9.3)	9.9 (8.4–14.4)	0.007	
Breast centroid to midline	Left	7.7 (7.2–8.9)	8.6 (8.1–10.2)	0.04	3.0 (1.5–3.4)
	Right	7.0 (6.2–7.7)	8.7 (7.3–9.4)	0.004	

*Values are expressed as median (range)*.

In total, the akimbo position was associated with an increase in median separation distance of 2.8 and 3.0 cm for the nipple and breast centroid, respectively. Moreover, larger breast volumes were correlated with larger arm position-related changes in breast (Pearson’s *r* = 0.74, *p* = 0.06) and nipple (*r* = 0.85, *p* = 0.02) separation.

### Breast Dosimetric Analysis

Table 3 illustrates that the akimbo arm positioning significantly decreased the breast mean dose (*p* = 0.02), V5 (*p* = 0.04), V10 (*p* = 0.02), and V15 (*p* = 0.04), but not V20 (*p* = 0.20), roughly demonstrating at least a twofold numeric improvement in each parameter. Of note, three out of the six female patients had mediastinal/axillary disease, and the effect of arm positioning in those patients was less pronounced. In this subset, the mean breast dose (median value) in akimbo versus arms raised conditions was 466 cGy and 619 cGy (*p* = 0.38). Similarly, there were no differences in breast V5 (*p* = 0.30), V10 (*p* = 0.30), V15 (*p* = 0.30), and V20 (*p* = 0.81).

**Table 3:** **Breast dosimetry in relation to arm positioning**.

Breast laterality	Technique	Mean dose (cGy)	V5 (%)	V10 (%)	V15 (%)	V20 (%)
Left	Raised	503.2 (223.1–1343.0)	27.2 (10.2–68.3)	21.1 (8.2–61.4)	16.8 (6.5–56.0)	7.5 (2.3–43.5)
	Akimbo	194.7 (143.2–1147.2)	9.8 (5.7–66.5)	6.9 (4.2–54.6)	5.5 (3.3–42.9)	3.1 (1.7–26.7)
Right	Raised	658.7 (492.3–751.2)	39.8 (28.7–42.3)	28.9 (18.2–32.6)	24.6 (14.0–29.0)	15.9 (2.5–20.8)
	Akimbo	422.3 (190.3–565.1)	21.4 (9.0–32.9)	15.8 (5.4–22.0)	13.3 (3.8–18.2)	7.6 (2.4–12.0)
*p*-Value	Raised vs. Akimbo	0.02	0.04	0.02	0.04	0.20

*Values are expressed as median (range). cGy, centiGray; V*n*, volume of organ (cm^3^) receiving at least *n* Gy*.

### Cardiopulmonary OAR Analysis

Table 4 depicts dosimetric parameters for the heart and lungs, for which there were no statistical differences between groups. Additionally, there were no arm position-related changes in relative heart or lung position with respect to the reference point (*p* > 0.05 for all directions). The median total lung volume for the raised arm CT was 2819 (range, 1539–3308) cm^3^ vs. 3070 (range, 1314–5792) cm^3^ for the akimbo scan (*p* = 0.15). The median heart volume was 618 (range, 439–744) cm^3^ vs. 569 (range, 396–815) cm^3^ on the arm-raised and akimbo scans, respectively (*p* = 0.984).

**Table 4:** **Cardiopulmonary dosimetry in relation to arm positioning**.

Technique	Mean heart dose (cGy)	Mean lung dose (cGy)	Lung V10 (%)	Lung V20 (%)
Raised	866.2 (363.6–1750.3)	755.3 (475.4–1341.4)	32.0 (19.4–63.9)	18.3 (7.9–34.3)
Akimbo	1237.8 (400.6–2136.4)	913.2 (470.9–1345.1)	41.5 (17.6–76.3)	21.2 (9.3–35.1)
*p*-Value, raised vs. akimbo	0.11	0.56	0.65	0.56

*Values are expressed as median (range). cGy, centiGray; V*n*, volume of organ (cm^3^) receiving at least *n* Gy*.

## Discussion

There have been multiple high-quality reports published regarding the development of breast cancer after mediastinal RT for HL; a primary theme therein emphasizes the utmost importance of measures to decrease breast doses as low as reasonably achievable (8–12). This is the first known study that quantitates breast doses and spatial parameters based on arm positioning, noting that akimbo positioning decreases breast doses and increases breast spatial separation, particularly in cases without axillary disease. This may be of importance to academic and community practices that may position patients at the discretion of the individual physician, largely owing to a dearth of data such as that reported herein.

It is noteworthy that there were no significant differences in craniocaudal breast position between techniques; rather, by far, the dominant mechanism of movement is in the medial–lateral directions. Hence, though arms raised positioning could be theoretically appealing for patients with appropriately located mediastinal tumors that could pull breast tissue upwards, this is, in fact, not the case. It is possible that the use of a wingboard versus alpha cradle (which may necessitate differential amounts of arm abduction) could change this notion, but there is no evidence for this presently. That cardiopulmonary doses were different (albeit statistically insignificant) between groups warrants further study. Though differences could result from positioning, breathing technique, and anatomical causes, larger sample sizes are required to confirm whether these differences persist.

A notable strength of this work is the relatively homogeneous patient treatment per the contemporary COG AHOD0031 protocol; all patients in this study were treated in the akimbo position. It should be mentioned, however, that the protocol does not allow multiangle three-dimensional conformal radiation therapy, intensity-modulated RT, or proton beam therapy (PBT), contemporary modalities that could theoretically further reduce breast doses compared to the AP/PA technique specified by the COG protocol. Moreover, it is also important to note that these patients were all treated in the era of involved field RT, prior to the widespread institution and acceptance of more limited RT treatment volumes in HL, such as the currently recommended involved site radiation (ISRT) (13). Hence, though our dosimetric results may not be applicable to these modalities/techniques, our spatial analysis of breast/nipple separation distance as a function of arm positioning still applies. Similarly, though no statistical differences in breast dosimetric parameters were observed in patients with mediastinal and axillary diseases, akimbo positioning still offers spatial advantages that could translate into dosimetric numerical differences possibly deducible with larger sample sizes. Similar work in patients treated with other modalities and other subgroups is hence needed as an extension of this study. For instance, larger breast volumes may stand to benefit to a greater degree from akimbo positioning.

The recent rise in PBT to treat not only pediatric cancers but also many other neoplasms (14–18) has necessitated greater data to support its use. Pediatric malignancies are one of the most agreed-upon indications of PBT, and the lower integral dose provided may limit occurrence of secondary, radiation-induced neoplasms. Application to pediatric HL cases is certainly noteworthy, as decreased cardiopulmonary and breast doses with AP PBT beams may aid in achieving lower incidences of late effects to these areas, albeit with virtually no long-term data to date.

The major limitation to our study is the use of involved field planning; recently, involved site RT has become the standard of care in this patient population. However, the AHOD0031 protocol is a contemporary protocol that was published recently in 2014. Many community practices and other countries still use involved field RT, and the results of this study are thus most applicable to these scenarios. The lack of heterogeneity corrections in this protocol is also noteworthy and results in higher estimated lung doses. Next, the finding of non-statistical differences between left and right nipples/centroids to midline may be related to individual positioning within immobilization frames, excursion of the arm during either positioning technique, and/or unforeseen patient rotation. Other limitations include not only the retrospective nature but also the relatively small sample size. Further research is hence needed to corroborate these conclusions, but our statistically significant breast dosimetry findings in patients with mediastinal (±cervical) disease cannot be discounted. Though cervical disease could impact breast doses, the magnitude is likely low, compared with the presence of axillary lymphadenopathy. Our non-uniform use of DIBH is another limitation, but studies have demonstrated that its use results in negligible changes to breast dose (19, 20).

## Conclusion

Taken together, this is the first report to quantitatively conclude that arm positioning in pediatric patients with HL undergoing mediastinal irradiation is substantially important to decrease breast doses. Further research using larger sample sizes and modern, conformal treatment modalities would also be of great interest in order to further expand this realm.

## Author Contributions

KD and CL conceived the project and performed data collection and analysis. VV, AB, and NB wrote and edited the manuscript. All authors approved the manuscript.

## Conflict of Interest Statement

The authors declare that the research was conducted in the absence of any commercial or financial relationships that could be construed as a potential conflict of interest.
